# A Bacterial Disease of Animals

**Published:** 1892-04

**Authors:** Henry G. Pyle


### A BACTERIAL DISEASE OF ANIMALS.

By Henry G. Pyle, D. V. S.

The so-called "Corn Stalk" Disease.

The rearing of domesticated animals is one of our principal industries in this prosperous country of ours. Anything materially interfering with the animal industry, becomes at once a matter of much importance to us, as veterinarians, as well as to the entire stock-producing world, and the consumers of meat in all parts of the world in which American produced meat is consumed. We have to-day, and have had for many years among our herds, a disease ill understood by veterinarians and stockmen as well. This disease is most commonly known as the "Corn Stalk" disease. This disease naturally, so far as I am aware, only attacks animals consuming foliage of corn-stalks. This disease had specially been prevalent in the States of Nebraska, Kansas and Iowa, though many cases had been reported from other States in the corn-producing sections of our country. The report of loss from this disease during the winters of 1889 and '90, in Nebraska, was from the following counties: Adams, 700 head of cattle, valued at $15,000, and fifty horses valued at $5,000. Cass cattle to the value of $3,500. Franklin animals valued at $4,000. Howard, $1,500. Karnay, $1,500. Hamilton, $3,600. Paurner, $2,000. Seward, $1,100. Webster, $4,000. Harlem, $1,500. Merrick, 1,000 head of cattle valued at $18,000. Furicas, no less than 5 per cent. of the cattle were lost. In Adams County 100 head of cattle and 20 horses were placed in a stalk field of 30 acres; in four weeks 63 head of cattle and five horses were dead. These animals were furnished salt each morning and allowed a millet stack and four acres of meadow to feed upon. In Kansas in counties as follows: Allen, 100 horses and a few cattle. Clay, 500 head of cattle valued at $7,000, and 25 horses valued at $800. Cloud, 200 head of cattle. Ellsworth, 2 per cent. of cattle. Geary, 300 head of cattle valued at $5,000, and 30 head of horses $1,000. Graham, $3,000 worth of cattle. Kingman, 100 head of cattle. Norton, $3,000 worth of cattle and $500 worth of horses. Osborne, $4,000 worth of cattle. Phillips, 1,000 head of cattle. Rice, 800 head of cattle. Smith, 500 head of cattle and 25 head of horses. Rooks, $4,000 worth of cattle and $800 worth of horses. In Iowa counties: Monroe, cattle $300, and horses $1,200. Shelby, 1,200 head of cattle valued at $12,000. Two counties in Missouri lost $4,750 worth of cattle. In Indiana, Pulaski County reports loss of 60 head of cattle valued at $1,200, and 30 horses valued at $2,200; small losses from other counties in same State. Small losses are also reported from many other corn-producing States. I had the unpleasant duty of inspecting a herd thus diseased in Central Illinois this winter, in which there was only a small per cent. of loss suffered.

This disease is usually seen when susceptible animals are first turned into the corn stalk fields, late in the fall, or in the early winter months, for to gather what feed remains in the field. It has been positively proven that it is not the consumption of the stalks which causes the disease, as many persons claim, by the presence of the wood-like substance in the food interfering with digestion, especially if sufficient water and salt is not furnished the animal during the ingestion of such material; no doubt this substance, as other indigestible material, may cause indigestion, and some symptoms and post-mortem appearances common to the so-called "Corn Stalk" disease. Dr. F. S. Billings claims this disease to be due to a vegetable parasite, a belted germ, neither a coccus nor bacillus, but much resembling each in form, which will be more fully described later.

Smutty corn, corn attacked by *Ustilago-maidis*, has been claimed by many persons to be the direct cause of this disease. Smut is a vegetable parasite, its fibers penetrate the tissue of a corn stalk, commencing when the stalk is very young, finally forming the large black masses of spores so easily recognized.

Some few years ago a Mr. Gangee, in three weeks, fed to two cows forty-two pounds of this smut with no ill effect. Smut is found in all corn fields each year, though the "Corn Stalk" disease is found only in certain fields. Mr. Gangee's and other experiments show there to be no question between the disease in question and smut.

Another cause frequently claimed to produce "Corn Stalk" disease is a lack of sufficient amount of salt and water furnished the animal, during digestion of food containing much dry, woody, fibrous material, as dry corn stalks, they gaining this idea from the appearance of the omasum of the ox, upon post-mortem examination, by finding the contents of the stomach very dry and much compact, a condition which may be found upon the examination of this stomach, after death, from any high febrile disease; this is an erroneous idea that the disease is simply an indigestion. Another fact is, the contents of the omasum does not always present this dry, compact condition so much spoken of. Water is often found in the Rumen, upon post-mortem examination, even if the contents of the omasum is found dry and compact in the "Corn Stalk" disease, thus showing it to be not from an insufficient amount of water. The salt theory is unworthy of comments at length, but I must say that animals have died from the "Corn Stalk" disease when salt was furnished the animals each morning regularly. Animals have died from "Corn Stalk" disease in fields through which streams of fresh water ran at all times. At times this disease has been known as Murrain, but as the term Murrain denotes no special disease it will not be further considered. The cause of "Corn Stalk" disease is the presence in the animal system of a specific micro-organisms, first discovered and described as the cause of the "Corn Stalk" disease by Dr. F. S. Billings, of Nebraska, in 1888. In 1889, Dr. Thomas J. Burrill, of Campaign, Ill., described "a bacterial disease of corn," since which time Dr. Billings has claimed this bacteria described by Dr. Burrill to be identical with his germ of the "Corn Stalk" disease; comparing the two germs in cultivation, I doubt their identity, though recognize their great similarity in growing. On slides they present no marked difference, to me, in appearance.

The "Corn Stalk" disease is a non-contagious septicæmic disease. The micro-organism, called by Dr. Billings a bacillus, resembles most a bacillus, which apparently does not find its primary origin in a diseased animal, nor in material from such an animal, but is an exogenous, infectious disease. The Billings germ is an oval, belted one, apparently a non-spore bearer, varying much in form and dimensions under various conditions. These germs reproduce only by subdivisions, so far as is at present known. The growth of these germs is very rapid in and upon many artificial cultivating substances, both fluid and solid. The growth will take place at the ordinary laboratory temperature, but grow more rapidly if maintained at blood heat. Upon neutralized, sterilized potatoes (cut surfaces), the germs thus grown produce a growth of light, grayish-white colored material, slightly elevated, with uneven edges.

Upon the whites of hard-boiled eggs, inoculated with these germs, produce a growth of clear, light, yellowish-colored colonies, the edges of which rise higher than the centre of the growth. Upon plain ager-ager standing tubes, the growth of these germs is pearly white in color, the growth is principally upon the surface of the medium, does not liquify the medium. In and upon beef-infusion gelatine needle-point cultures, these germs upon the surface forms a firm, thin growth, spreads very slowly, the puncture also grows slowly, the growth is pearly gray in color, the medium remains firm. Upon gelatine plates, they grow in colonies, more round and some irregular in shape, the growth is partly beneath the service of a gray color, the medium remains solid. The growth of this germ in neutral beef-infusion is very rapid, causing the contents of the flask to become clouded, but lighter in color. A micro-germ forms on the surface, and a white precipitate forms. The end of. these germs are rounded, the length is about .00125 mm., their breadth is about .0007 mm. when measured in blood-stained mounts. The middle portion of the body of a mature germ presents a semi-transparent appearance, bounded by two ends of darker appearance. In the hanging drop of fluid from the beef-influsion culture, the germs can be seen in rapid motion; they turn end for end a few times, then change their movements to a peculiar locomotion by raising and lowering themselves in the fluid. During their development they vary much in form and dimensions; when first separated from the mature form they most resemble a coccus in form, but gradually, though rapidly, change to the mature form. At times two or three mature germs may be linked together, appearing as a chain, but the marks of separation may be plainly seen. These germs stain readily with Ziehl's solution (Fuchsin), or aniline-blue.

EXPERIMENTS.—Dr. Billings has made many successful experiments in connection with this disease. He has succeeded in causing death in susceptible animals by feeding corn stalk leaves and tops of corn supposed to be diseased with the "bacterial disease of corn" which Dr. Burrill has so completely described. From animals thus destroyed, Dr. Billings obtained pure cultures of the "Corn Stalk" disease. With these cultures he destroyed susceptible animals by inoculation, which presented lesions of the "Corn Stalk" disease, from which animals he again obtained pure cultures.

No. 1. Upon Jan. 27, 1892, at 3.30 P. M., I inoculated one six months' old Jersey bull calf with pure culture of a "Corn Stalk" disease, in beef-infusion. The injection was intervenous (jugular vein), of 9 c. c. of fourteenth generation of the germ. At the same time one white rabbit, female, (previously inoculated with other germs to no ill-effect) was inoculated with one-third c. c. of same cultures as was above animal. The rabbit died on Jan. 29, 1892, at 5 o'clock P. M.; the autopsy was held immediately after death; the usual lesions of "Corn Stalk" disease in animals thus killed was found, from which animal pure cultures of the germs of "Corn Stalk" disease was obtained. At the time of inoculation of the calf his temperature was 39.25 C. (rectal). This calf was found dead Jan. 29, 1892, A. M.; soon after death the autopsy was held, at 10 o'clock A. M., same day. No external markings, except a non-supprurating wound at place of inoculation, which presented the appearance of a healthy wound, the eges of which were held together by granulation; lymph glands were swollen, clouded and of an increased redness in color, small intestines, serosa swollen, reddish slate color, vessels injected, some spots of increased redness; large intestines, darker than normal, with congested spots present; lungs slightly congested throughout each lobe; spleen, normal; heart, anti-mortem clot present; blood darker than normal, having but little tendency to coagulate; bladder and contents normal; liver slightly swollen, cut surface presented an increase of fat corpuscles, acinus distended, hemoragic spots were seen throughout the capsule, gall bladder distended; kidneys slightly swollen, capsule not adhering, cortes of increased redness, ingorgement of straight vessels, nedullar substance of increased redness, nesenteric vessels ingorged; stomach, rumen presented a normal condition; reticulam mucosa slightly congested; omasm in normal condition; abomasum mucosa inflamed and gathered into an increased number of folds; rectum, mucosa slightly darker than normal in color. In the same pen with calf No. 1 was confined a Jersey calf of same age throughout the experiment, receiving the same treatment as calf No. 1, except the inoculation. This calf presented no symptoms of ill-health. In the blood of calf No. 1 was found the germs of the "Corn Stalk" disease in great abundance. On Jan. 29, the lungs, liver and spleen of calf No. 1 were fed to two hogs in one pen; all the organs were placed in the pen at one time. The two hogs were a lot of six bought at the same place at the same time. These hogs were common ones, one half grown. On the morning of Feb. 3, one of the two hogs was found dead, having died during the previous night; autopsy 11 A. M., same day. No external markings, no effusion in abdominal or thoracic cavities; lymph glands somewhat swollen and clouded; small intestines, serosa swollen and clouded, vessels injected, generally of a lead and gray color, interposed with patches of diffused redness; large intestines, serosa same as small, convolutions filled with a reddish yellow coagulated material, closely attached to underlaying tissues.

Organs of thoracic cavity, normal; spleen somewhat swollen, dense and cut surface clowded and somewhat dry. Liver intensely swollen at base, edges rounded, general surface of a brownish-gray-red color; acinus distended. The brownish-gray-red color spoken of, generally interrupted by acinus of same, of a yellowish color, cut surface corresponded thereto, dry and anæmic; kidneys slightly swollen, capsule not adhering; cortex anaemia, yellowish-gray-red in color, with ingorgement of straight vessels; medullary surface bright red in color.

Stomach: Mucosa excessively swollen and gathered into a deep ruage; the base of fundas mucosa entirely engorged, of bright red color, and covered with a yellowish-gray corrugated coating, loosely attached; mucosa of pelvic portion intensely swollen, viscid coating of a yellowish-leaden color. Mucosa of duodumen and jejunum swollen, of a general yellowish-gray-leaden color, interrupted by defuse patches of a defused redness. Mucosa of ilium still more swollen, af a slightly dark red color. Peyers patches swollen. Large intestines, mucosa swollen; transverse folds of a dirty yellowish-leaden-gray color, interrupted by patches of a reddish color. The surface of the entire micosa was covered with a more or less closely attached dirty yellowish-gray material, which presented a defuse granulated appearance; at the closely attached places, the removal of this granulated membrane exposed an ulcerated surface. Mucosa of rectum swollen; of a dirty-yellowish-gray color. Contents of stomach and bowels semi-fluid. This hog blood was used to innoculate three ager-ager tubes, from which pure cultures of the germs of "Corn Stalk" disease were obtained in the onusual form and number, as obtained from the blood of bovines having died from this disease. From these ager-ager tubes, plain gelatine tubes were innoculated, obtaining the same germs in their usual form. From the same hog blood two beef infusion flasks were innoculated, in which the germs in pure form were present, the hog in pen with the one that died, fed the same as the one that died, soon afterwards presented symptoms of ill health, and remained ill for more than two weeks, during which time it lost flesh, but had little appetite, but recovered; perhaps not having received so much of the diseased and disease-producing meat; the attack of the disease was not so severe as in the one which died. The remainder of the six hogs remained in good health throughout this time. On January 31st, a small amount of the same virus as calf No. 1 was innoculated with, was placed in one thousand grammes of beef-infusion, which produced the usual changes in the contents of the flasks. On February 22nd, the contents of these flasks were fed (taken with great relish without any loss of the fluid) to one six months old calf (which had previously been innoculated with another disease to no ill effect) at the time of feeding; the temperature of this calf was normal, but soon the temperature was elevated and remained above normal for a number of days, during which time the calf was stupid, appetite decreased, and in an unhealthy condition; in three weeks it again gained its normal condition.

"Symptoms of the "Corn Stalk" disease in cattle: Death may occur before you know the animal is ill, or the animal may remain ill for a number of days, then recover from the illness. The animal is often found prostrate, tympanetic with increased respiration, semi-comatose ; in this condition they usually die in a short time ; they always appear as in much pain, and have an unsteady locomotion. When prostrate they arise with great difficulty, if able to rise. Constipation is usually present. The other symptoms are those usually found present in febrile diseases. The symptoms may much vary in the different animals affected in the same herd. Treatment, so far, has been followed with little success, though benefit may be obtained by use of salines febrafuges if given early. Prevention is the best treatment; the herd should be removed from the infected field as soon as an animal is found ailing, to uninfected quarters, and each member of the herd should have a dose of epsom salts or other salines.

Among the large number of beef cattle exported to Europe from this country during the past few months were found upon examination at the abbatoirs, lesions, especially in the lungs of the animals of, to European veterinarians, a strange disease. After a few experiments and investigations, Prof. Nocard, of France, at once suspected the disease to be the "Corn Stalk" disease, and announced his thoughts as such. The English veterinarians, in whose hands animals or portions of organs of ailing animals perchance to fall, soon caused much commotion by some of them announcing the disease to be contagious pluro-pneumonia; others gave it a name of some less malignant ailment. Our foreign friends need have no fear of procuring contagious pluro-pneumonia in their herds from our beef or other cattle, as the much-dreaded disease, contagious pluro-pneumonia, is unknown in our beef-producing sections. And as to the " Corn Stalk " disease, no fear should exist, as this disease is only local and a non-contagious one. The animals which usually die are the two-year-olds in best condition. What animals are susceptible to this disease are to be yet found out. No mention, so far as I know, has before been made to swine being susceptible animals to this disease.

My many thanks are especially due to Dr. F. S. Billings, Director of the Patho-Biological Laboratory of the State University of Nebraska, as well as to Dr. Thomas J. Burrill, Horticulturist and Botanist of the University of Illinois for assistance, material and conveniences furnished me in gaining substance for this paper.

